# NCRAD: A Critical Resource for the Alzheimer's Disease and Related Dementias Research Community

**DOI:** 10.1002/alz70856_107593

**Published:** 2026-01-09

**Authors:** Kelly N. Nudelman, Kristen A. Russ, Michael C. Edler, Kelley M. Faber, Jeffrey L. Dage, Jason S. Meyer, Adrian L Oblak, Tatiana M. Foroud

**Affiliations:** ^1^ National Centralized Repository for Alzheimer's Disease and Related Dementias (NCRAD), Indianapolis, IN, USA; ^2^ Department of Medical and Molecular Genetics, Indiana University School of Medicine, Indianapolis, IN, USA; ^3^ Indiana Alzheimer's Disease Research Center, Indianapolis, IN, USA; ^4^ National Centralized Repository for Alzheimer's Disease and Related Dementias (NCRAD), Indianapolis, IN, USA; ^5^ Stark Neurosciences Research Institute, Indiana University School of Medicine, Indiana, IN, USA; ^6^ Department of Neurology, Indiana University School of Medicine, Indianopolis, IN, USA; ^7^ Stark Neurosciences Research Institute, Indiana University School of Medicine, Indianapolis, IN, USA

## Abstract

**Background:**

The National Centralized Repository for Alzheimer's Disease and Related Dementias (NCRAD) supports the etiology, early detection, and therapeutic development for Alzheimer's disease and related dementias (ADRD). One of the goals of NCRAD is to continue to offer high‐quality biobanking, biospecimens, standardized ADRD biomarkers, and support for investigators utilizing cutting‐edge methods and assays to advance ADRD research.

**Method:**

NCRAD currently funds sample processing and banking for ADRD studies, and for AD Research Centers (ADRCs), supports generation of *APOE* genotype, plasma‐based ADRD biomarkers, and supplemental GWAS data. Rigorous quality control ensures the highest quality biospecimens are processed, banked, and distributed, including: sample tracking; DNA and RNA quality measurements; hemoglobin contamination assessment; DNA fingerprinting to assess sample quality and identity; and whole genome sequencing (WGS) data generated for all banked induced pluripotent stem cell (iPSC) lines. Genetic and biomarker data is shared with data repositories including the National Institute on Aging Genetics of Alzheimer's Disease Data Storage Site (NIAGADS) and the National Alzheimer's Coordinating Center (NACC).

**Results:**

NCRAD currently banks samples for more than 80 studies, including samples from >130,000 participants with sample types including DNA, RNA, whole blood, plasma, serum, CSF, brain tissue, stool, peripheral blood mononuclear cells (PBMCs), lymphoblast cell lines, fibroblasts, and iPSCs. More than 15,000 samples are from paired visits with more than one sample type available, and many studies bank samples from longitudinal participant visits. The repository distributed 20,000 uniform sample collection kits and received, processed, and stored more than 200,000 new aliquots in 2024. Approximately 440,000 sample aliquots have been distributed to nearly 300 researchers thus far. To date, over 1,000 publications have been generated using NCRAD samples and data.

**Conclusion:**

NCRAD has played a key role in development of best practices, protocol development, and uniform sample collection for ADRD studies. NCRAD continues to support cutting‐edge research in genetics, genomics, and biomarker research and, more recently, implementation of the Alzheimer's Association Revised Criteria for diagnosing AD by making plasma biomarker data broadly available for ADRC participants. As such, NCRAD has and continues to play a vital part in the advancement of translational ADRD research.

